# *Cryptosporidium* spp. prevalence in the general population in Guinea: first large-scale screening study[Fn FN1]

**DOI:** 10.1051/parasite/2024070

**Published:** 2024-11-13

**Authors:** Timothé Guilavogui, Nausicaa Gantois, Jérémy Desramaut, Fode Ibrahima Cissé, Salif Cherif Touré, Bakary Luther Kourouma, Cristian Preda, Magali Chabé, Eric Viscogliosi, Gabriela Certad

**Affiliations:** 1 CNRS, Inserm, CHU Lille, Institut Pasteur de Lille, U1019-UMR 9017-CIIL-Centre d’Infection et d’Immunité de Lille, Université de Lille 59000 Lille France; 2 Unité d’Appui à la Gestion et la Coordination des Programmes, Ministère de la Santé et de l’Hygiène Publique Conakry BP 585 Guinea; 3 Hôpital National Ignace Deen CHU de Conakry, Laboratoire de Parasitologie Conakry BP 1263 Guinea; 4 Centre de Santé Anastasis Conakry BP 1032 Guinea; 5 Délégation à la Recherche Clinique et à l’Innovation, Groupement des Hôpitaux de l’Institut Catholique de Lille, Lille Catholic University 59000 Lille France

**Keywords:** *Cryptosporidium* spp, Molecular epidemiology, Transmission, Zoonosis, Guinea, Africa

## Abstract

*Cryptosporidium* is a leading cause of diarrheal mortality in children in Africa and Asia. Despite the public health significance of this parasite, its molecular epidemiology and circulation in Guinea remain poorly understood. Therefore, this study aimed to determine the prevalence and genotype distribution of *Cryptosporidium* in the Guinean general population. To achieve this, fecal samples were collected from 834 individuals, both with and without digestive disorders, at two hospitals in Conakry. The presence of the parasite in the stool samples was detected using nested PCR targeting the SSU rDNA gene, followed by sequencing of the PCR products for genotyping of the isolates. The PCR-based prevalence was 0.12% for the whole cohort, and 0.2% among adults. The low frequency of *Cryptosporidium* observed in the current study is thus consistent with the prevalence of this parasite already reported in certain other African countries. The species identified in the positive samples was *Cryptosporidium hominis*. This study is the first to report the prevalence of *Cryptosporidium* in the general population of Guinea. Given the potential of this parasite to cause life-threatening diarrhea, further studies are needed to clarify the epidemiology of *Cryptosporidium* in this country.

## Abstract (fr)

*Cryptosporidium* est une cause majeure de mortalité diarrhéique chez les enfants en Afrique et en Asie. Malgré l’importance de ce parasite en matière de santé publique, son épidémiologie moléculaire et sa circulation en Guinée restent mal comprises. Par conséquent, cette étude visait à déterminer la prévalence et la distribution génotypique de *Cryptosporidium* dans la population générale guinéenne. Pour y parvenir, des échantillons fécaux ont été collectés auprès de 834 individus, avec et sans troubles digestifs, dans deux hôpitaux de Conakry. La présence du parasite dans les échantillons de selles a été détectée à l’aide d’une PCR imbriquée ciblant le gène SSU ADNr, suivie d’un séquençage des produits de PCR pour le génotypage des isolats. La prévalence basée sur la PCR était de 0,12 % pour l’ensemble de la cohorte et de 0,2% chez les adultes. La faible fréquence de *Cryptosporidium* observée dans la présente étude est donc cohérente avec la prévalence de ce parasite déjà rapportée dans certains autres pays africains. L’espèce identifiée dans les échantillons positifs était *Cryptosporidium hominis*. Cette étude est la première à rapporter la prévalence de *Cryptosporidium* dans la population générale de Guinée. Étant donné le potentiel de ce parasite à provoquer une diarrhée potentiellement mortelle, d’autres études sont nécessaires pour clarifier l’épidémiologie de *Cryptosporidium* dans ce pays.

## Introduction

Parasites of the genus *Cryptosporidium* are intracellular protozoa belonging to the phylum Apicomplexa. This genus comprises species that infect the gastrointestinal tract of many vertebrates, including humans [8]. *Cryptosporidium* species cause a cosmopolitan emerging opportunistic infection with a considerable impact on immunocompromized hosts such as HIV/AIDS patients, in whom cryptosporidiosis can become chronic or even lethal [32]. Additionally, cryptosporidiosis is considered the second leading cause of infant mortality due to diarrhea [23, 24].

Oocysts are the propagative form of *Cryptosporidium*, becoming infectious immediately upon excretion. These oocysts contaminate the environment, posing a significant threat as they facilitate transmission between humans and animals [3]. These forms are resistant to typical disinfectants and water chlorination, and can remain viable and infectious for several months [37].

The infection is transmitted via the fecal–oral route, by ingestion of oocysts present in fecally contaminated water or food, or by contact with an infected host. However, infection can also occur in immunocompetent individuals, either sporadically or in epidemic outbreaks, typically resulting in self-resolving, uncomplicated diarrhea [8]. The species most commonly infecting humans are *C. parvum* and *C. hominis*, although infections by other species such as *C. meleagridis*, *C. felis*, *C. canis*, *C. ubiquitum*, and *C. cuniculus* have been reported, mainly in immunocompromized patients [38]. Currently, no effective specific treatment is available for cryptosporidiosis [3].

Cryptosporidiosis has a significant impact on public health, particularly in low-income countries. Most African countries are considered low-income or lower-middle-income economies according to World Bank definitions [37]. Of the 31 countries worldwide classified in the lowest income group, 24 (77%) are in Africa [37]. One of the first studies to examine the impact of *Cryptosporidium* in an African country was carried out in Guinea-Bissau. In this study, *Cryptosporidium* infection was identified in 239 episodes of diarrhea (7.4%) out of 3,215 reported in 205 children; in addition, the parasite was associated with high mortality in children under 2 years of age [30]. Twenty years later, the Global Enteric Multicenter Study (GEMS), which included a cohort of over 20,000 children, provided the first worldwide estimates of the impact of cryptosporidiosis across different age groups in Africa and East Asia [24]. In particular, cryptosporidiosis was identified as the second leading cause of diarrheal mortality in children aged 12–24 months in Africa and India. In these regions, transmission of the disease, primarily caused by *C. parvum* and *C. hominis*, is mainly human-to-human. The risk factors associated with infection include high population density, poor hygiene conditions, lack of sewage treatment, and young age [24, 50].

*Cryptosporidium* infection in children under 5 years of age has been associated with 44.8 million diarrheal episodes and 48,300 deaths worldwide [23]. *Cryptosporidium*-associated diarrhea mortality is particularly high in sub-Saharan Africa among children under 5 years of age. For instance, 23,300 (48%) *Cryptosporidium-*related deaths in this age group were reported in Nigeria (*n* = 18,900) and the Democratic Republic of the Congo (*n* = 4,900) [18].

Furthermore, it has been shown that damage to intestinal epithelial cells caused by the infection significantly harms children’s gut health, impairing nutrient absorption and leading to stunted growth, reduced neurocognitive development, and other long-term consequences [35]. Considering the downstream effects of stunting associated with cryptosporidiosis, it is estimated that the prevalence of this parasite could be 2.5 times higher than previously thought [37].

The prevalence of cryptosporidiosis is expected to increase by up to 70% in some regions of the world by 2050 due to urbanization and climate change, making the prevention and treatment of this parasitic infection crucial, especially for immunocompromized individuals and children [3].

Despite the public health significance of *Cryptosporidium*, its molecular epidemiology and circulation in Guinea are not well understood. Therefore, the aim of the current study was to characterize the prevalence of *Cryptosporidium* in the general population of Guinea.

## Material and methods

### Ethics approval

The present study was approved by the National Ethics Committee on Health Research (CNERS) of Guinea (reference number 170/CNERS/20; approval date: 24 December 2020). It was conducted in accordance with the Declaration of Helsinki III and the International Ethical Guidelines for Biological Research Involving Human Subjects. Participants were thoroughly informed about the research project prior to enrollment, and written informed consent was obtained from each adult participant or from the parents or guardians of minor participants.

### Questionnaire survey

A standardized questionnaire was designed to collect information about each participant, including gender, age, place of residence, source of drinking water (drilling, tap or mineral water), contact with domestic animals, and presence of digestive symptoms (i.e., diarrhea, abdominal pain, vomiting, bloating, and constipation). A participant was considered symptomatic if at least one of the five specified digestive disorders was present. All subjects’ data were fully anonymized.

### Sampling

This survey was conducted in West Africa, Republic of Guinea and precisely in Conakry (geographical coordinates: latitude 9°32′16″ N, longitude 13°40′38″ W), the capital and largest city of the country, with an estimated population of approximately 2,300,000. Participants were recruited among patients seeking care for different disorders, with or without gastrointestinal symptoms in two hospitals of the city: the National Hospital Ignace Deen (NHID) (*n* = 534 patients) and the Confessional Health Center Anastasis (CHCA) (*n* = 300 patients) located in the South and North of Conakry, respectively. Sampling was completed in two periods: the first one between January and March 2021. This period of the year corresponded to the dry season under a tropical monsoon climate. At this season, almost no precipitation falls in Conakry and the daily mean temperature reaches 25–27 °C. The second collection was performed in July 2022, corresponding to the wet season; the daily mean temperature reaches 30–32 °C. For each patient, one stool sample was collected at each hospital during routine standard care.

Around 2 g of fresh stools was collected in 2 mL of 2.5% potassium dichromate (w/v in water) (Sigma Life Sciences, Saint Louis, MO, USA) in a sterile tube. All samples were stored at 4 °C and then transported to the Pasteur Institute of Lille (France) for further analysis.

All data on patients are available in Supplementary Table 1.

### Molecular detection of *Cryptosporidium*

DNA was extracted from approximately 200 mg of fecal samples using a NucleoSpin 96 Soil Kit or NucleoSpin Soil Mini Kit (Macherey-Nagel GmbH & Co. KG, Düren, Germany), according to the manufacturer’s instructions. This kit is useful for *Cryptosporidium* detection considering that it contains a bead beating step, which helps to break the oocyst walls, improving molecular detection rates. DNA was kept at −20 °C until use. A nested PCR targeting the 18S rRNA gene was performed as previously [49] with slight modifications (the analytical sensitivity of this technique in our laboratory for the detection of *Cryptosporidium* DNA from 5 μL of serial 10-fold 18S rRNA plasmids diluted in a final volume of 50 μL is 10 copies, which is equivalent to at least 1 oocyst [9]). The external primers used were 5′-TTCTAGAGCTAATACATGCG-3′ (forward) and 5′-CCCATTTCCTTCGAAACAGGA-3′ (reverse). The internal primers used were 5′- GGAAGGGTTGTATTTATTAGATAAAG-3′ (forward) and 5′-AAGGAGTAAGGAACAACC TCCA-3′ (reverse). The first PCR mixture was prepared in a final volume of 50 μL as follows: 10 μL of DNA, 1x HotStarTaq Plus buffer, 2 mM MgCl_2_, 0.4 μM for each primer, 0.4 μM dNTP each and 1.5 U HotStarTaq Plus DNA polymerase (QIAGEN Inc., Hilden, Germany). The conditions for the PCR were as follows: 94 °C for 5 min, followed by 40 cycles of 94 °C for 45 s, 65 °C for 45 s, and 72 °C for 1 min. The post-extension was completed at 72 °C for 5 min. The second PCR mixture was prepared in a final volume of 50 μL as follows: 2 μL of the primary PCR product, 1x HotStarTaq Plus buffer, 1.5 mM MgCl_2_, 0.4 μM for each primer, 200 μM dNTP each and 1.5 U HotStarTaq Plus DNA polymerase. The nested PCR conditions were the same for both rounds. Nested PCRs were performed in a Gene touch Hangzhou BIOER Thermal Cycler (Hangzhou Bioer Technology Co., Ltd, Hangzhou, China).

### DNA sequencing and analysis

Positive PCR products were purified and the amplicons were sequenced on both strands (Sanger technology), using the forward and reverse primers of the nested PCR by the company Genoscreen (Pasteur Institute of Lille, Lille, France). Comparisons with similar sequences of *Cryptosporidium* available on the NCBI (http://www.ncbi.nlm.nih.gov/BLAST/, accessed in August 2023) and CryptoDB servers (https://cryptodb.org/cryptodb/app/workspace/blast/new, accessed in July 2024) were performed using the basic local alignment search tool (BLAST). To consider the sequences analyzed in this study as the same *Cryptosporidium* species when compared to references, the identity value should be in the range of 98–100% sequence similarity. The nucleotide sequence identified in this study was deposited in GenBank under the accession number PQ101122.

### Descriptive statistics

Frequencies and their 95% associated confidence intervals (95CI) for the risk factors were calculated.

## Results and discussion

Demographic characteristics of the study population are detailed in Table 1. Among the 834 patients followed-up at the two hospitals included in this study, 48.56% were female and 51.43% were male. Participants’ ages ranged from 1 year to 83 years, with a median of 26 years (IQR: 11–36). The age distribution was as follows: 7.43% were children aged 0–5 years, 35.25% were aged 6–18 years, and 57.31% were 19 years and older. The majority of participants (75.77%, 632/834) resided in Conakry, while the remaining participants were from towns in the suburbs of the city. In this cohort, 680 individuals (81.53%) were classified as symptomatic, having experienced at least one of the 5 selected digestive symptoms as follows: abdominal pain was the most common symptom (603/834, 81.53%), followed by constipation (185/834, 22.18%), diarrhea (58/433, 13.4%), and vomiting (18/433, 4.2%). None of the patients reported bloating. The remaining 67 participants (13.4%) were asymptomatic at the time of the study (Table 1)*.* Ten patients out of 834 reported being immunosuppressed, and from this group, four persons declared that they were HIV+. We did not have access to data to confirm the HIV infection status of volunteers included in the study*.*

**Table 1:** Demographic characteristics of the study population.

Demographic and clinical data	Frequency (%)	CI (95%)
Gender		
Male	429/834 (51.43)	[48.04; 54.82]
Female	405/834 (48.56)	[45.17; 51.95]
Age (years)		
≤5	62/834 (7.43)	[5.57; 9.10]
6–18	294/834 (35.25)	[32.00; 38.50]
≥18	478/834 (57.31)	[53.95; 60.67]
Living in Conakry	632/834 (75.77)	[72.86; 78.67]
Living in other areas	202/834 (24.22)	[21.31; 27.12]
Drinking water source		
Exclusively mineral water	36/834 (4.31)	[2.93; 5.69]
Different sources (tap, spring, mineral)	798/834 (95.68)	[94.30; 97.05]
Contact with animals		
Yes	122/834 (14.63)	[12.23; 17.02]
No	712/834 (85.37)	[82.97; 87.76]
Unwashed fruits and vegetables consumption		
Yes	17/834 (2.04)	[1.09; 2.30]
No	817/834 (97.96)	[97.00; 98.91]
Large household size (more than seven members)		
Yes	331/834 (39.6)	[36.28; 42.92]
No	503/834 (60.31)	[56.99; 63.63]
History of parasite infection		
Yes	156/832 (18.75)	[16.10; 21.39]
No	676/832 (81.25)	[78.60; 83.90]
Travel out of Guinea		
Yes	20/834 (2.40)	[1.36; 3.44]
No	814/834 (97.60)	[96.56; 98.64]
River bathing		
Yes	15/834 (1.80)	[0.90; 2.70]
No	819/834 (98.20)	[97.30; 99.10]
Presence of symptoms	680/834 (81.53)	[78.89; 84.16]
Abdominal pain	603/834 (72.30)	[69.26; 75.33]
Diarrhea	83/834 (9.95)	[7.91; 11.98]
Vomiting	29/834 (3.48)	[2.24; 4.72]
Constipation	185/834 (22.18)	[19.36; 24.99]
Bloating	0/834 (0)	–
Immunodepression		
Yes	10/834 (1.19)	[0.46; 1.94]
No	824/834 (98.80)	[98.06; 99.54]
HIV+		
Yes	4/834 (0.48)	[0.01; 0.94]
No	830/834 (99.52)	[99.05; 99.99]

*Cryptosporidium* infection was identified in only one sample collected in the dry season, representing a prevalence of 0.12% for the entire cohort, and 0.2% when considering only adults. Even though seasonality is considered a driver for cryptosporidiosis in tropical countries with an increasing prevalence of *Cryptosporidium* during the rainfall season [45], no *Cryptosporidium* cases were detected during this period in the current study.

A recent meta-analysis about the prevalence of intestinal parasitosis in Guinea reported two studies on *Cryptosporidium* prevalence among HIV patients [18]. To our knowledge, this is the first study to provide a recent estimate of the presence of *Cryptosporidium* spp. in the general population of this country. The only positive case was a 27-year-old non-immunocompromized patient who did not experience diarrheal symptoms but did report occasional abdominal pain. This individual lived in a household composed of 5 adults and 10 children.

Interestingly, living in a large household of more than seven members has been described as a risk factor for *Cryptosporidium* infection. This is likely due to the fact that, in larger households, the risk of person-to-person transmission is potentially increased [2]. Other behaviors increasing the risk for *Cryptosporidium* infection such as animal contact, consumption of unwashed vegetables, poor drinking water quality, lack of toilet facilities, travel out of Guinea, or river bathing were not identified.

Table 2 summarizes surveys for *Cryptosporidium* conducted among the general population in Sub-Saharan Africa, revealing prevalences ranging from less than 1% to 32.4%. The low frequency of *Cryptosporidium* observed in the current study is thus consistent with the ≤1% prevalence of this parasite already reported after using molecular diagnostic methods in other African countries among the general population, such as Madagascar, Ethiopia, Kenya, Tanzania, and Cote d’Ivoire [6, 13, 15, 16, 22] (Table 2).

**Table 2:** *Cryptosporidium* frequency in the general population in Sub-Saharan African countries according to different studies*.*

Country	Year*	Age group (years)	Population	Presence of symptoms	*Cryptosporidium* Frequency No. positive/No. samples (%)	Diagnostic methods	Molecular markers	*Cryptosporidium* species	References
Nigeria	2007	Adults	People in the community	NR	204 (rural: 29.7, urban: 19.4)	Formol-ether and microscopy (modified Ziehl–Neelsen staining)	NA	ND	[21]
Zambia	2007	Adults	Farmers	Not digestive disorders	18/289 (6)	ELISA and nested PCR	SSU-rRNA and HSP70	*C. parvum/C. hominis*	[43]
Ethiopia	2010	1–45	People seeking medical attention, including HIV patients	Digestive disorders	79/1034 (7.6)	Microscopy (Ziehl–Neelsen staining), nested PCR	COWP, SSU-rRNA and GP60	*C. hominis*, *C. parvum*	[1]
Zimbabwe	2010	All ages	People in the community	NR	19/300 (6.3)	Microscopy (Ziehl–Neelsen staining)	NA	ND	[29]
Cameroon	2011	Adults	People in the community	NR	1/35 (2.9) (considering only controls)	Immunoassay	NA	ND	[36]
Uganda	2012	<75	People in the community (human volunteers)	Not digestive disorders	35/108 (32.4)	Nested PCR	COWP	*C. parvum/C. hominis*	[40]
Madagascar	2014	NR	People in the community	NR	1/120 (0.8)	PCR-RFLP	SSU-rRNA	*C. suis*	[6]
Mali	2015	18–30	European soldiers	Digestive disorders	3/51 (5.7)	Multiplex PCR	NR	*C. parvum*	[17]
Sudan	2015	1–80	Inhabitants of 2 rural areas considering only >20 years old	NR	34/247 (13.3)	Microscopy (Ziehl–Neelsen staining)	NA	ND	[42]
Tanzania	2015	NR	People in the community	Digestive disorders	8/185 (4.3)	PCR-RFLP	SSU rRNA	*C. hominis*	[34]
Ethiopia	2015	< 80	People seeking medical attention	Digestive disorders	1/92 (1.1)	Microscopy (Ziehl–Neelsen staining), nested PCR assay	GP60	*C. hominis* IbA9G3	[15]
Burkina Faso	2015	All ages	People seeking medical attention	Digestive disorders	77/291 (26.5)	Formol-ether concentration and modified Ziehl–Neelsen staining	NA	ND	[41]
Cote d’Ivoire	2015	>12 months	People in the community	Digestive disorders	5/121 (4.13)	Antigen detection RDT, multiplex reverse transcriptase (RT-) PCR	NR	ND	[4]
Kenya	2016	2–81	People in the community	NR	?/796 (<1)	Real-time PCR	NR	NR	[13]
Tanzania	2016	All ages	People in the community	NR	2/174 (1.1)	Real-time PCR	SSU-rRNA	ND	[16]
Ghana	2017	Adults	Livestock farmers	NR	8/95 (8.4)	Real-time PCR	SSU-rRNA	*C. parvum*	[46]
Mozambique	2018	>17	People seeking medical attention, excluding immunosuppressed people	Digestive disorders	9/108 (8.3)	PCR-RFLP	SSU-rRNA	*C. parvum*, *C. hominis*, *C. felis*	[7]
Madagascar	2020	All ages	People in the community	NR	4/49 (8.16)	Immunoassay	NA	NA	[44]
Ethiopia	2021	6–66	People seeking medical attention, excluding immunosuppressed people	Digestive disorders	23/95 (24.2)	Microscopy, nested PCR and real-time PCR	SSU-rRNA and GP60	*C. parvum* and *C. hominis*	[20]
Madagascar	2021	18–36	Household contacts	NR	4/52 (7.69)	Nested PCR	SSU-rRNA	*C. hominis*	[14]
Gabon	2021	6–25	Household contacts	NR	16/79 (20.25)	Nested PCR	SSU-rRNA	*C. parvum*, *C. hominis*, *C. felis*	[25]
Tanzania	2021	7–30	Household contacts	NR	16/114 (14.03)	Nested PCR	SSU-rRNA	*C. parvum* and *C. hominis*	[25]
Ghana	2021		Household contacts	NR	11/105 (10.48)	Nested PCR	SSU-rRNA	*C. parvum*, *C. hominis*, *C. felis*	[25]
Ethiopia	2022	>6	Farmers	NR	10/113 (8.8)	Sheather sugar solution and Microscopy (modifed Ziehl–Neelsen staining)	NA	NA	[5]
Cote d’Ivoire	2024	1–75	People in the community	Not digestive disorders	3/259 (1.2) (considering only controls)	Multiplex real-time PCR	NR	ND	[22]
Mali	2024	1–56	People in the community	Not digestive disorder	26/547 (4.8) (considering only controls)	Multiplex real-time PCR	NR	ND	[22]
Ethiopia	2024	Adults	People seeking medical attention	NR	14/122 (11.5)	Microscopy (Ziehl–Neelsen staining)	NA	NA	[19]

*Year of publication; NR: Not reported; NA: Not applicable; ND: Not determined; table modified from [45].

In other studies, among healthy individuals without symptoms in Qatar, Malaysia, and Taiwan, prevalences around 5% were observed [26, 27, 39]. A previous molecular survey carried out in the Akkar district of North Lebanon revealed a *Cryptosporidium* prevalence of 4% among symptomatic, immunocompetent adult patients [33]. Other studies indicated that *Cryptosporidium* prevalence can exceed 70% or more in symptomatic patients with diarrhea [45]. Additionally, results from various African studies have shown that *Cryptosporidium* prevalence in immunocompromized patients, particularly those with HIV and low CD4+ cell counts, can reach nearly 50% [45].

The *Cryptosporidium* species identified in the positive sample was *C. hominis*, an anthroponotic species. The corresponding sequence matched 100% with a *C. hominis* sequence previously reported in Uganda and formerly identified as *C. parvum* genotype 1 (AF481962). This sequence was identified among hospitalized children (0–5 years old) with diarrhea and a significant association between *Cryptosporidium* infection and malnutrition including stunting, being underweight, and wasting was observed [47]. Several authors agree that anthroponotic and zoonotic transmissions are responsible for *Cryptosporidium* infection in Africa, with a predominance of *C. hominis* and of an anthroponotic subtype of *C. parvum* [23, 37].

Interestingly, various hypotheses have been proposed to explain the low prevalence of *Cryptosporidium* in certain areas of Africa: 1) a lower frequency of *Cryptosporidium* has been found in African urban settings compared to rural ones [31]. Such is the case of the current study in which 75.77% of the population was from Conakry. This difference is likely due to urban populations having better access to improved water sources and sanitation healthcare facilities. Moreover, in urban settings, there is less potential for zoonotic transmission of *Cryptosporidium* from livestock or wildlife. Additionally, rural populations tend to be poorer than their urban counterparts, which affects their level of hygienic practices; 2) the lack of *C. parvum* observed in native cattle breeds in some African regions might reduce zoonotic cryptosporidiosis transmission to humans [28]; 3) early-life exposure to *Cryptosporidium* could limit future infections; and 4) genetic variations in the population may contribute to make individuals more or less susceptible to specific infections [37]. For instance, a genetic variant within protein kinase C alpha (PRKCA) was identified and associated with an increased risk of cryptosporidiosis in the first year of life. Interestingly, the highest frequencies of this genetic variant were observed in East Asian populations where the prevalence of cryptosporidiosis is high. In contrast, this allele was less common in West Africa [48].

Limitations of our study include the examination of only one stool per patient. Indeed, intermittent oocyst excretion is recognized and for this reason, 3 stool samples are considered the optimal number necessary to detect the microorganism [11]. In addition, in the current work, we focused on the study of the general population, including a majority of persons older than 5 years (92.56%). Therefore, this cohort may not fully represent the disease burden, suggesting that a high number of asymptomatic or mild diarrheal cases are probably missed. In addition, a previous meta-analysis about the prevalence of intestinal parasitosis in Guinea showed a trend towards a higher prevalence in studies conducted in the community compared to those performed at health care centers [18], as is the case in the current research.

In conclusion, this is the first study reporting the prevalence of *Cryptosporidium* in the general population in Guinea. Although the overall prevalence is low, the presence of the parasite remains a public health concern due to its potential to cause severe diarrhea in certain patients. Interestingly, the prevalence of HIV, one of the risk factors of *Cryptosporidium* infection, is estimated at 1.5% among people aged 15–49 in this country. This prevalence is higher among homosexuals (9.8%) and drug users (3.6%) [12]. Further longitudinal studies are essential to elucidate the molecular epidemiology and pathogenesis of *Cryptosporidium* infection, as well as to understand the roles of host and environmental factors in susceptibility, immune response, and clinical outcomes of cryptosporidiosis [10]. These studies will help clarify the dynamics of infection, improve diagnostic and treatment strategies, and inform public health interventions to better manage and prevent the impact of this parasitic disease.

## Supplementary material

The supplementary material of this article is available at https://www.parasite-journal.org/10.1051/parasite/2024070/olm. *Supplementary Table 1*. *Cryptosporidium* spp. prevalence in general population in Guinea: study data.
